# Enhancing healthcare efficiency to achieve the Quadruple Aim: an exploratory study

**DOI:** 10.1186/s13104-020-05199-8

**Published:** 2020-07-31

**Authors:** Bengt B. Arnetz, Courtney M. Goetz, Judith E. Arnetz, Sukhesh Sudan, John vanSchagen, Kyle Piersma, Fredric Reyelts

**Affiliations:** 1 Department of Family Medicine, College of Human Medicine, Michigan State University, Grand Rapids, MI USA; 2 Mercy Health, Grand Rapids, MI USA; 3 Trinity Health, Livonia, MI USA

**Keywords:** Efficiency, Quadruple Aim, Quality improvement, Productivity, Team-based care

## Abstract

**Objective:**

Healthcare is battling a conflict between the Quadruple Aims—reducing costs; improving population health, patient experience, and team well-being—and productivity. This quasi-experimental pilot study tested a 2 week intervention aimed to address the Quadruple Aims while improving productivity. Participants were 25 employees and their patients in a primary care clinic. One provider and their team implemented an efficiency-focused intervention that modified work roles and processes focused on utilizing all team members’ skills as allowable by applicable licensure restrictions. The five remaining providers and their teams comprised the reference group, who continued patient care as usual. Study outcomes were measured via provider/staff and patient surveys and administrative data.

**Results:**

In total, 46 team surveys and 156 patient surveys were collected. Clinic output data were retrieved for 467 visits. Compared to the reference team, the intervention team performed better in all Quadruple Aims and productivity measures. The intervention group offered 48% more patient slots than the average reference team. These preliminary results support the feasibility of introducing substantial process changes that show promising improvement in both the Quadruple Aims and productivity. A larger-scale study over a longer time period is needed to confirm findings and examine feasibility and cost-effectiveness.

## Introduction

In 2014, the Quadruple Aim—adapted from the widely-accepted Triple Aim [1]—was suggested as a framework to optimize healthcare system performance. The framework encompasses reducing costs, improving population health and patient experience, with a new fourth domain: healthcare team well-being [2]. These performance dimensions can be applied to far-reaching, crucial healthcare challenges, such as reducing the massive rates of burnout present in healthcare workers [3] and combating rising healthcare costs [4]. These foci are crucial for healthcare quality, yet healthcare systems must also consider other factors. Reimbursement for care provided in the United States is based on productivity, i.e., work relative value units (wRVU), despite a shift towards value-based care by the Centers for Medicare and Medicaid Services [5]. Most private insurers mimic this productivity-based reimbursement strategy [6].

Thus, healthcare systems are facing 2 daunting yet seemingly opposed challenges: striving to achieve the goals proposed in the Quadruple Aim [2] while increasing productivity [7]. There are an increasing number of forces that create demands on providers’ performance and cognitive load. These include expectations of physicians to generate wRVU by seeing more patients [8,9], suboptimal design of the EHR (electronic health record) [7], shifting patient/consumer expectations of the provider-patient relationship, and a rapidly increasing alternative primary care sector, e.g., walk-in clinics, urgent care, concierge medicine, and online offerings. Many physicians spend hours of overtime completing EHR and other administrative tasks [7]. Despite these pressures, physicians are also dedicated to providing quality care to their patients [2]. These burdens trickle down within teams, creating a stressful environment wherein team members must work with administrative tasks instead of focusing on patient care [2,7]. These competing demands contribute to the burden that healthcare professionals are experiencing today, likely encouraging moral distress [10] and burnout [3] and creating a cycle that makes it even harder to provide high-quality care. Despite this, interventions tend to target one specific problem rather than comprehensively targeting the challenges experienced in primary care. For example, interventions such as mindfulness and stress management are often used to improve the well-being of the healthcare team. While these intervention strategies can foster improvement related to the targeted issue, they often fail to address the root causes of stress and burnout [11], and may be a temporary fix for organizational problems that will eventually return.

Practice change, and the incorporation of research evidence into routine clinical practice can be extremely challenging for healthcare workers. Considering the many time-related demands and pressures that healthcare team members face in their daily work, the added responsibility of changing routine care practice or workflow can seem tedious and unimportant. Yet, obtaining buy-in from clinic staff is crucial for implementation success [12]. The relative advantage of the intervention and its compatibility with perceived needs likely enhance buy-in [13], so interventions that simplify work processes and reduce work stress may be more effectively implemented.

**Fig. 1:**
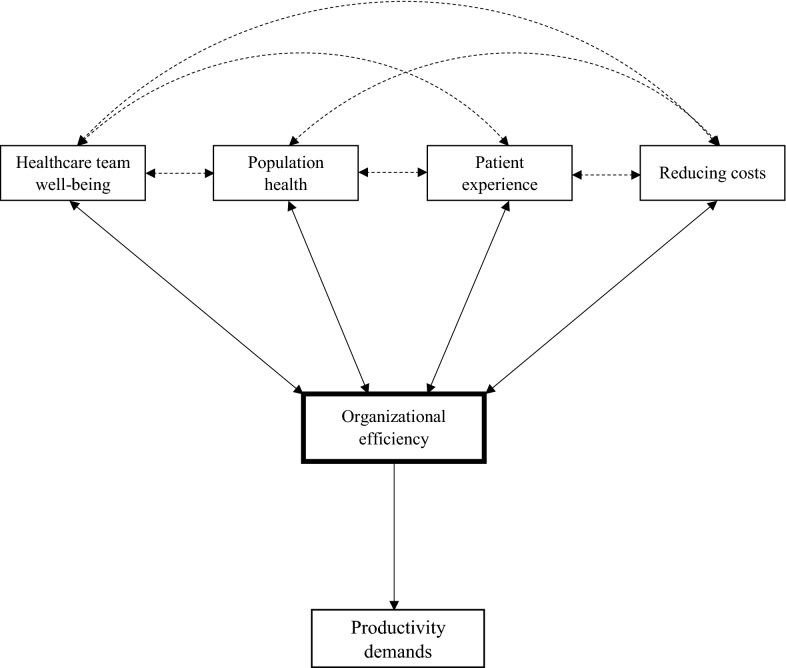
Theoretical modeling linking all Quadruple Aims to organizational efficiency and productivity

An intervention is needed that comprehensively targets the numerous demands faced in primary care delivery. Using theory informed by prior research [14–17], we posit that enhancing healthcare efficiency can simultaneously address these demands without requiring additional resources (Fig. 1). To our knowledge, no previous intervention has primarily targeted efficiency for quality improvement. Optimal clinic efficiency is achieved when appropriate resource use creates an environment that promotes teamwork and skills development while protecting against work stress, burnout, and dissatisfaction. This enables team members to provide high-quality care and a positive patient experience [16]. While a *productivity* focus requires outcomes and puts pressure on individual providers to create results, an *efficiency* focus is related to process changes and requires organizational change. Thus, theoretically, a focus on efficiency should allow personnel to achieve performance measures while improving workplace well-being since resources and processes are more effective, and team members are working at the top of their license (using the most advanced skills they were trained/educated for). Below, we present results from a 2 week pilot test of an efficiency-focused intervention in a single primary care clinic.

## Main text

### Methods

#### Study design

This quasi-experimental study took place over 2 weeks (10 working days) in early 2019 in a family practice clinic consisting of 25 employees, including 6 providers. The intervention team consisted of one 1.00 full-time equivalent (FTE) provider, two 1.00 FTE medical assistants (MA), and one 0.25 FTE registered nurse (RN), the existing staffing model of the clinic. The remaining five providers and their teams (n = 21 employees) comprised the reference group.

#### Efficiency-focused flow intervention to energize care teams (EFFECT)

Informed by prior studies [16] and clinical experience [18,19], it was hypothesized that the intervention team would simultaneously meet all Quadruple Aims and clinic productivity by improving clinic efficiency. The team set clear goals for the intervention: increase visit capacity by 50% and complete all administrative and electronic health record activities within the 8 h workday. The intervention, EFFECT, focused on revising patient flow to allow preplanning of work tasks (e.g., tests and procedures), scribing, and optimizing skills utilization of all team members. Daily team “huddles” and between-visit “touch-bases” were incorporated in order to plan, communicate, and assign responsibilities. All appointments were scheduled for 20 min. One additional acute visit was allotted each hour. Necessary lab draws and vaccinations were completed as part of the intake process by a MA or RN, who also completed all EHR and administrative duties immediately before, during, or after the visits. The provider was thus able to focus fully on the patient encounter with no computer-related tasks.

The reference team continued work as usual with the existing scheduling model: 25 min for acute patient needs and 50 min for physicals and chronic needs.

#### Measures

##### Aim 1: reducing costs

Cost reduction was measured by visit capacity, i.e., the total number of slots available for patient visits per full-time equivalency (FTE) provider. Increasing the number of slots allows for the distribution of fixed costs over more visits.

##### Aim 2: population health

The number of HIV screenings completed per day per FTE provider was used as a proxy for population health procedures administered. This measure showed the largest gap in meeting clinical guidelines.

##### Aim 3: healthcare team well-being

Healthcare team members completed a 14-question survey about their experience four times: 1 day pre-intervention, days 1 and 10 of the intervention, and 7 days post-intervention. Participants responded to statements, e.g., “I feel fulfilled at work”, adapted from the Quality Work Competence survey [20], each item using a 0–10 visual analogue scale (VAS) response format.

##### Aim 4: patient experience

At check-out, patients were asked to complete a 16-question paper survey about their experience. Some questions reflected those asked in the team surveys, e.g., “There is a positive work environment in this clinic”, while others were adapted from the Consumer Assessment of Healthcare Providers and Systems (CAHPS) [21]. Patients responded using a 0–10 VAS for each item.

##### Productivity

Total wRVU and visits completed during the study were calculated for each FTE provider, as well as mean wRVU per visit. Intervention provider productivity was compared before and during the intervention using mean wRVU.

##### Efficiency

Patients and the healthcare team, respectively, rated efficiency (“My visit was efficient”, “Our clinic runs efficiently”) using a 0–10 VAS.

#### Analysis

Statistical analysis was conducted using IBM SPSS, V.25, 2018 (IBM Corp, Armonk, NY). Independent samples t-tests were used to analyze group differences in wRVU and visit capacity/visits completed. For the intervention provider, one sample t-tests were used to compare mean wRVU per visit during the intervention versus 6 months pre-intervention. Pearson’s correlations were used to examine relationships between both patient and healthcare team-rated efficiency and the survey variables. Significance was set at a p-value of < 0.05. Parametric tests were confirmed using non-parametric tests.

### Results

During the intervention, the clinic completed a total of 495 visits (200 intervention provider, 295 reference providers; Table 1). Clinic output data was collected for 94% of visits (n = 467, 184 intervention, 283 reference). A total of 46 survey responses were collected from the healthcare team (average response rate 61.11%), and 156 from patients (31.52%). There were no statistically significant between- or within-group differences in patient or healthcare team survey variables, including efficiency (data not shown).

**Table 1:** Clinic outputs, intervention vs. reference team during the intervention

	Intervention team (1 provider)	Reference team (5 providers)	
*Clinic output*	*Mean (st.dev)*	*Mean (st.dev)*	*t*_*df*_
Total wRVU over study period^a^	267.90 (NA^b^)	143.74 (51.17)	t_465_ = − 40.82***
Mean wRVU per visit	1.46 (NA)	1.44 (0.08)	t_465_ = − 2.62**
Visit capacity^*a*^	241 (NA)	162.74 (15.51)	t_41_ = − 28.99***
Visits completed^*a*^	200 (NA)	77.18 (24.90)	t_41_ = − 28.34***
Clinic outputs, intervention provider before vs. during intervention			
	Pre-Intervention	Intervention	
*Clinic output*	*Mean (st.dev)*	*Mean (st.dev)*	*t*_*df*_
wRVU per visit	1.34 (0.82)	1.46 (0.43)	T_183_ = 3.68***

**p < 0.01, ***p < 0.001

^a^Providers adjusted to 1.00 Full Time Equivalency to allow for across team comparison

^b^Not applicable

#### Aim 1: reducing costs

The intervention team had a significantly higher visit capacity than the reference team (Table 1).

#### Aim 2: population health

The intervention team completed significantly more daily HIV screenings than the reference group (M = 2.30, SD = 1.77 vs. M = 0.31, SD = 0.56; t = − 3.51, p = 0.006), and completed more HIV screenings during the intervention (n = 23) than during the entire 6 months prior (n = 22).

#### Aim 3: healthcare team well-being

Healthcare team-rated efficiency correlated positively with team-rated professional fulfillment and skills utilization, and negatively with stress (Table 2).

**Table 2:** Correlation of patient and team experience variables to efficiency

Variable	Pearson’s *r*
Healthcare team experience	
Professional fulfillment	0.532***
Skills utilization	0.496**
Stress	− 0.336*
Patient experience	
Rating of doctor	0.633***
Willingness to recommend clinic	0.442***

*p < 0.05, **p < 0.01, ***p < 0.001

#### Aim 4: patient experience

Patient-rated efficiency correlated positively with patient ratings of their doctor and willingness to recommend the clinic (Table 2).

#### Productivity

The intervention provider generated significantly higher mean wRVU compared to reference providers. The intervention provider also generated significantly more total wRVU and completed more visits than reference providers and increased mean wRVU per visit during the intervention compared to pre-intervention (Table 1).

### Discussion

EFFECT was associated with improvement in all Quadruple Aims and productivity. Efficiency ratings were highly and significantly correlated with better patient and team experiences, including lower work stress. By realigning professional tasks and substantially changing work processes to better utilize the skills of all team members, the intervention team was able to schedule appointments with twice as many patients and double total wRVU compared to the reference group. This was accomplished without adding staff or provider time, or requiring overtime. Thus, our hypothesis that focusing on efficiency would result in more effective work habits and processes, thus allowing the same performance and improved workplace well-being, was supported. Performance measures were improved while work stress was reduced. This suggests that stress within a typical primary care setting could potentially be addressed by revising how care is delivered, including making sure that all personnel, including physicians, utilize all their skills and experience as allowable under their licenses.

By increasing visit capacity, EFFECT also increased patient access. Improved access to primary care has been shown to reduce spending and utilization of more costly specialty, emergency, and inpatient services [22,23]. Revising clinic processes allowed for identification of quality gaps before the visit, so that the intervention team could close gaps in population health quality measures [24,25]. While HIV screenings were our primary population health outcome, anecdotal reports suggest that the intervention team closed or decreased several other quality gaps (immunization, diabetes mellitus indicators, mammography, etc.), regardless of visit type. The reorganized work process may have allowed the intervention team to have more intentional, productive time with each patient.

Anecdotally, the healthcare team reported having positive impressions of EFFECT. The physician reported higher work-life satisfaction, being able to adequately focus on his patients, and feeling more prepared for a complicated patient. The medical assistants were also satisfied with the intervention. They felt more knowledgeable about the patients they served, making their EHR tasks easier. However, the MAs did feel that it was a lot more work and may warrant higher compensation. Especially for these team members, the transition to working at the top of their license involved more responsibilities and demands. While they generally viewed EFFECT positively, the change process was somewhat taxing.

### Conclusions

This preliminary study suggests that many of the challenges being discussed in current primary care discourse, such as burnout, stress, access limitation, and revenue concerns, are malleable by addressing work roles and processes through enhanced skills utilization. Results suggest that interventions targeting clinic efficiency have the potential to foster broad improvements in outputs, including the Quadruple Aims and productivity. A strength of this preliminary study was the combined use of stakeholder self-report measures with clinical administrative data. Nevertheless, a larger-scale study over a longer time period, including cost-effectiveness analysis is warranted. Moreover, the feasibility of implementing these clinical process changes on a larger scale in operationally-strained primary care clinics needs to be further evaluated.

## Limitations

This was a small, preliminary study of a previously untested, albeit theoretically-informed intervention. It was conducted at a single site, utilized a small sample size, and was conducted over a limited (2 week) time period. Results are preliminary and may not be generalizable to other primary care clinics.

## Data Availability

The datasets used and/or analysed during the current study are available from the corresponding author on reasonable request.

## Abbreviations

wRVU: Work relative value units
EHR: Electronic health record
FTE: Full-time equivalent
MA: Medical assistant
RN: Registered nurse
EFFECT: Efficiency-focused intervention to energize care teams
HIV: Human immunodeficiency virus
VAS: Visual-analog scale
CAHPS: Consumer assessment of healthcare providers and systems
